# Higuchi’s fractal dimension, but not frontal or posterior alpha asymmetry, predicts PID-5 anxiousness more than depressivity

**DOI:** 10.1038/s41598-019-56229-w

**Published:** 2019-12-23

**Authors:** Tame N. J. Kawe, Shabah M. Shadli, Neil McNaughton

**Affiliations:** Department of Psychology, University of Otago, Dunedin, New Zealand

**Keywords:** Diagnostic markers, Anxiety, Depression

## Abstract

Depression is a major cause of health disability. EEG measures may provide one or more economical biomarkers for the diagnosis of depression. Here we compared frontal alpha asymmetry (FAA), posterior alpha asymmetry (PAA), and Higuchi’s fractal dimension (HFD) for their capacity to predict PID-5 depressivity and for the specificity of these predictions relative to PID-5 anxiousness. University students provided 8 or 10 minutes of resting EEG and PID-5 depressivity and PID-5 anxiousness questionnaire scores. FAA and PAA had no significant correlations with the measures at any electrode pair. There were distinct frontal and posterior factors underlying HFD that correlated significantly with anxiousness and with each other. Posterior HFD also correlated significantly with depressivity, though this was weaker than the correlation with anxiousness. The portion of depressivity variance accounted for by posterior HFD was not unique but shared with anxiousness. Inclusion of anxiety disorder patients into the sample rendered the frontal factor somewhat more predictive than the posterior one but generally strengthened the prior conclusions. Contrary to our predictions, none of our measures specifically predicted depressivity. Previous reports of links with depression may involve confounds with concurrent anxiety. Indeed, HFD may be a better measure of anxiety than depression; and its previous linkage to depression may be due to a confound between the two, given the high incidence of depression in cases of severe anxiety.

## Introduction

Depression is now the major cause of global health disability^1,2^; with a 12-month prevalence of over 5%^3,4^. The combination of depression with anxiety^5^ is particularly disabling^6,7^. The key problem for both diagnosis and treatment is that the fundamental biological nature of these disorders is unknown – and so biomarkers for diagnosis are not available^8,9^.

Non-invasive scalp electroencephalography (EEG) is temporally and spatially complex and so is a potentially rich and economical source of neural biomarkers for depression, with alpha asymmetry^10–12^ and fractal dimension^13–15^ already linked to depression. Here we ask if their predictions of trait depression (depressivity) are independent and/or overlap prediction of trait anxiety (anxiousness).

Alpha asymmetry (AA) is the difference in alpha band (8–12 Hz) power of homologous scalp electrodes between the right minus left hemisphere. Low AA reflects relatively less right alpha power and has been linked to trait measures of anxiety^16^, depression^17^ and negative affect^18,19^. But results with AA and depression have been mixed^20,21^ due to differences in gender^22,23^, participant populations^11,24^, pharmacological treatment response^10,12,25^, and comorbid anxiety^26^. Further, frontal AA (FAA) appears to be distinct functionally as well as in neural location from that at posterior sites (PAA)^27^, although FAA and PAA have both been linked to depression^28–30^.

Fractal dimension (FD) is a non-linear measure of a signal’s complexity. Increased EEG synchrony results in its reduction. This has been observed in epileptic seizures (which exhibit increased synchronous oscillations and a reduction of arrhythmic activity^31,32^) and under anaesthesia (with HFD demonstrating efficacy in estimating depth of anaesthesia^33^). In contrast, complexity increases are seen in manic phases of bipolar disorder^34^ and in the frontal lobes of schizophrenia patients^35^. Both bipolar disorder and schizophrenia feature disrupted synchronous activity^36,37^. Higuchi’s fractal dimension (HFD)^38^ is one method of calculating the FD of a time series and is applied in the current analysis. A small number of studies have assessed HFD in depressed populations^13–15,39^, with all finding it higher; suggesting that HFD may useful as a depression biomarker. However, HFD has not been tested for equivalence to AA, potential gender differences, or comorbidity of depression with anxiety.

FAA and PAA differ in terms of the brain areas generating them and HFD differs from both in reflecting complexity rather than power. We, therefore, predicted they would have unique (i.e. not shared) correlations, summating to predict depressivity. AA may relate more generally to negative affect than specifically to depressivity. We, therefore, also predicted that the AA measures (and possibly HFD as well) would correlate with anxiousness, potentially generating an interaction between the two, with anxiety and depression potentially moderating each other’s relationship to any of the EEG measures^26^.

Clinical depression is heterogeneous, medication affects EEG^25^, and separating anxiety and depression for clinical groups is difficult. We, therefore, tested participants using trait measures from the Personality Inventory of the Diagnostic and Statistical Manual of Mental Health Disorders – Fifth Edition (PID-5)^40^, which was developed for the identification of pathological traits. We used high PID-5 ‘depressivity’ as a proxy for clinical depression. The PID-5 is clinically derived, has good reliability and validity^41^, and scores can encompass both clinical and community samples^42,43^. We used the PID-5 ‘anxiousness’ scale in the same participant group as a proxy for clinical anxiety to allow direct comparison and included the trait scale from the Spielberger State-Trait Anxiety Inventory^44^ (STAI-T) to provide a general assessment of clinical morbidity. As with the PID-5 this scale allows us to compare its trait measure across clinical and general populations. Previous mixed results are likely due to method differences. We, therefore, initially tested the effect of different eye conditions (eyes open versus eyes closed) and bandwidths used in previous research (see Supplementary Material). As expected^22,23^, strong gender-specific effects were found and so females and males were analysed separately. Correlations between AA and trait scores were minimal. Posterior HFD was significantly correlated with PID-5 anxiousness and this partially generalised to PID-5 depressivity in a general sample. The general pattern of correlations remained the same when a clinically anxious sample was included. These correlations were non-specific and appear to relate equally to anxiousness and depressivity.

## Results

### AA does not vary significantly with depressivity or anxiousness

AA was calculated from the Fourier power spectrum (averaging log power for each of 3 alpha sub-bands: low 2 – 6–8 Hz; low 1 – 8–10 Hz; and high – 10–12 Hz) and then taking the right-left difference for each frontal and posterior electrode pair (frontal: Fp2-Fp1, AF4-AF3, F4-F3, F8-F7; posterior: P4-P3, P8-P7). Signed percentage of variance accounted for by each AA measure is displayed in Table 1. None reached statistical significance. Male participants show the expected pattern of negative correlations with depressivity in frontal electrodes and this persisted in the posterior electrodes. Female participants show primarily negative (but miniscule) correlations in frontal electrodes and positive correlations in posterior electrodes.

**Table 1:** Signed percent variance in depressivity and anxiousness accounted for by AA at each electrode, bandwidth and measure split by gender.

Measure	Pair	Female			Male		
		Low2	Low1	High	Low2	Low1	High
Depressivity	Af4-Af3	−1.0	−0.7	−0.1	0.0	−1.6	−9.0
	Fp2-Fp1	−0.7	−0.3	0.0	0.0	−1.8	−4.8
	F4-F3	0.1	0.1	0.8	0.0	−1.7	−7.4
	F8-F7	−1.4	−1.4	0.0	0.8	−0.6	−1.6
	P4-P3	5.4	3.5	0.1	−0.2	−9.4	−1.4
	P8-P7	1.5	4.4	0.4	1.2	−1.3	−0.6
Anxiousness	Af4-Af3	−0.7	−0.8	−0.1	0.5	0.0	−0.6
	Fp2-Fp1	−0.1	0.0	0.1	0.0	−0.6	−2.5
	F4-F3	0.6	0.3	1.0	0.4	0.2	0.0
	F8-F7	−4.0	−5.0	−2.0	3.1	0.7	0.0
	P4-P3	0.6	3.0	0.1	−0.7	−2.6	−2.4
	P8-P7	0.2	1.7	0.3	−0.6	−1.9	−0.6

### HFD predicts anxiousness

A high level of inter-correlation of HFD between channels was observed. Due to this, a principle components analysis was applied (see Supplementary Material). Two components with eigenvalues exceeding 1 were identified that represented distinct frontal and posterior components. Components appeared to be functionally identical across gender, although they differed in weighting. Female participants loaded heavily onto the posterior component while male participants loaded more heavily onto the frontal component. HFD scores were then combined into two measures reflecting the main components. Frontal (FP2, FPZ, FP1, F8, F4, FZ, F3, F7) and posterior channels (P8, P4, PZ, P3, P7). As the topography of the components did not differ by gender, further analysis did not split results by gender.

Frontal HFD and posterior HFD were input to a stepwise linear regression for each of the personality measures. Posterior HFD was identified as the strongest predictor for PID-5 anxiousness (r = 0.331, F(1,97) = 11.964, p < 0.001), PID-5 depressivity (r = 0.209, F(1,97) = 4.424, p = 0.038) and STAI-T (r = 0.262, F(1,97) = 7.154, p < 0.01). Frontal HFD significantly predicted PID-5 anxiousness (r = 0.239, F(1,97) = 5.892, p = 0.017) and STAI-T (r = 0.236, F(1,97) = 5.741, p = 0.018) although it did not significantly predict PID-5 depressivity. Forcing both HFD measures into regressions found that frontal HFD contributed no unique variance to the equation for any measure and so further analysis focussed on posterior HFD.

Anxiousness, Depressivity and STAI-T were then regressed against posterior HFD to determine whether posterior HFD correlates with unique variance in each or represents some common component in all three measures. The results are in Table 2. Almost all variance accounted for by STAI-T was shared with Anxiousness. All Depressivity variance was shared with STAI-T. This indicates that the components of STAI-T and Depressivity that correlate with posterior HFD are a common component shared with the Anxiousness scale, which had a unique component accounting for somewhat less than half of the total variance accounted for.

**Table 2:** Regression coefficients predicting Posterior HFD in the original sample expressed as percentage proportion of variance.

Measure	Zero Order	Part	Shared	Total
Anxiousness	10.98	6.93	4.05	11.29
Depressivity	4.36	0.31		
Anxiousness	10.98	5.15	5.83	12.02
STAI	6.87	1.04		
Depressivity	4.36	0.15	4.21	7.02
STAI	6.87	2.65		
Anxiousness	10.98	5.00	5.97	12.02
Depressivity	4.36	0.00	4.36	
STAI-T	6.87	0.73	6.14	

### HFD predicts anxiousness in a clinical sample

In response to comment on our original submission of this paper, we included a clinically anxious sample that we had available from a separate study. (A depressed sample is not available.) The variance of scale scores of the clinical and general population was compared. The clinical population displayed greatly reduced variance in PID-5 anxiousness (F(1,143) = 50.133, p < 0.00001), PID-5 depressivity (F(1,143) = 76.151, p < 0.00001), and STAI (F(1,143) = 138.456, p < 0.00001) scales. Due to the substantial narrowing of variance, the clinical sample was first analysed separately and then pooled with the general sample to provide the required variance for further analysis.

As expected, given the narrow variance, within the clinical sample alone HFD did not predict PID-5 depressivity (Frontal HFD: r(46) = 0.101, p = 0.506; Posterior HFD: r(46) = 0.063, p = 0.680) or PID-5 anxiousness (Frontal HFD: r(46) = 0.103, p = 0.496; Posterior HFD: r(46) = −0.032, p = 0.833). With the addition of the clinical sample to the general sample, there were modest decreases in percent variance accounted for and substantial increases in statistical significance. The frontal HFD component became the strongest correlate of all 3 scales despite the fact that the sample was predominantly female (who appeared to have a posterior bias in the original sample). Frontal HFD prediction was strongest with STAI-T (r = 0.291, F(1,143) = 13.212, p = 0.0004), followed closely by PID-5 anxiousness (r = 0.285, F(1,143) = 12.650, p = 0.0005) and then PID-5 depressivity (r = 0.243, F(1,143) = 8.946, p = 0.003). Results for posterior HFD reduced for PID-5 anxiousness (r = 0.270, F(1,143) = 11.209, p = 0.001), STAI-T (r = 0.190, F(1,143) = 5.378, p = 0.022) and PID-5 depressivity (r = 0.165, F(1,143) = 4.018, p = 0.047). Stepwise regression selected frontal HFD alone as the best predictor with posterior HFD predicting no unique variance. Thus, despite a shift in which factor was detected as the primary one, the general structure of the results remained the same.

Regressing the scale measures against frontal HFD found that predicted variance is shared across all measures (see Table 3). HFD correlates with a component that is shared by all 3 scales. In particular, as before, depressivity has no unique variance. Anxiousness retained a unique component of about one third of the variance accounted for when combined with depressivity. However, with the inclusion of STAI-T this dropped to less than a fifth of the variance accounted for.

**Table 3:** Regression coefficients predicting frontal HFD in the combined (original plus clinical) sample expressed as signed percentage of variance.

Measure	Zero Order	Part	Shared	Total
Anxiousness	8.13	3.17	4.95	9.06
Depressivity	5.89	0.94		
Anxiousness	8.13	1.52	6.61	9.99
STAI-T	8.46	1.85		
Depressivity	5.89	0.01	5.88	8.47
STAI-T	8.46	2.58		
Anxiousness	8.13	1.52	6.61	9.99
Depressivity	5.89	0.00	5.89	
STAI-T	8.46	0.92	7.54	

## Discussion

AA did not significantly predict either PID-5 anxiousness or PID-5 depressivity in either male or female participants. Our findings agree with a recent meta-analysis of frontal AA that found only a small and non-significant effect size^45^. An earlier meta-analysis had indicated significant effects^20^ although according to Gold, *et al*.^21^ this may have been due to publication bias skewing towards positive results. The present results suggest that AA does not have a strong relationship with the trait measures of either PID-5 depressivity or PID-5 anxiousness. Further testing, including a clinically depressed sample, would be desirable.

Posterior HFD, derived from averaged posterior channels, produced the strongest correlations with depressivity and with anxiousness in our original sample. However, its correlation with depressivity was not unique suggesting that it could be the result of the common tendency for depression to occur in the context of high anxiety. STAI-T overlapped almost completely with anxiousness, but not vice-versa. Anxiousness provided a unique contribution of slightly under half of the predicted HFD variance when combined with depressivity in the original analysis. But, when the clinical sample was included, anxiousness had only a modest unique contribution particularly when STAI-T was included as a predictor.

Elevated HFD in MDD patients has been consistently reported^13,15,39^ and posterior electrodes may have particular prominence^39^. There is limited previous EEG research investigating HFD in relation to anxiety. Correlation dimension, a related measure, has been observed to increase with severity of anxiety symptoms^46^. The change in HFD of treatment-resistant anxiety participants in response to ketamine does not appear to correlate with symptom improvement^47^. However, that study focused on within-patient changes in clinical outcomes over only a 2-hour time period and did not assess correlations between EEG and longer-term trait measures across a population. Overall, then, it is possible that HFD is directly linked to some aspect of trait anxiety and that previous reports of links with trait depression are secondary and due to comorbidity.

As noted in the introduction, the observed elevation in HFD could reflect a decreased EEG synchrony with increased trait anxiousness. As essentially all the HFD variance associated with depressivity and with STAI-T was shared with anxiousness (but not vice-versa), it is likely that the shared component is impaired synchrony linked to trait anxiety that has a common impact on the three measures. For frontal HFD this would be consistent with the links between trait anxiety (and anxiolytic drug action) and frontal midline theta^48^.

Whatever the causal basis of the observed variation in HFD, our results suggest that it, but not FAA or PAA, varies with changes in trait anxiousness, and that it has a weaker, consequential, relationship with depressivity. Given the variation in HFD with epilepsy, phases of bipolar disorder, and schizophrenia its relation, even to trait anxiousness, is unlikely to be specific.

## Methods

### Participants

Three independent samples were analysed in this study. The relaxation EEG data that are reported here from these samples have not been included in any publication previously.

Two were general samples that differed only in the order in which they received the relaxation test and a Stop Signal Task (SST) and which were pooled for analysis (see statistical analysis). The pool consisted of right handed participants (18–37 years old, mean = 21.60, SD = 3.34, recruited from Student Job Search. From a total of 101, 2 were excluded due to excessive artefacts in their EEG data, leaving 65 females and 24 males for data analysis. Participants with a current psychiatric diagnosis were excluded.

The third sample was a clinical group, consisting of 48 anxiety patients (18–56 years old, mean = 33.27, SD = 10.51), who received the relaxation test after the SST. These were not part of the original study design and were included, by request, during revision of the original submission. They were recruited from public health organisations located in Dunedin and Auckland and through a variety of advertisements including some in the online Otago Daily Times, supermarket advertisements, and Facebook. They reported chronic symptoms of anxiety but were excluded if they were currently on any pharmacological treatment for this, if they had a history of substance abuse, or if they had any neurological disorder. A MINI diagnostic examination, undertaken by a trainee clinical psychologist, found that all met the criterion for a DSM anxiety disorder. (The diagnoses included GAD, SAD, GAD with concurrent MDD, panic, anorexia, and PTSD). 1 was removed due to excess artefacts in their EEG, leaving 39 female and 8 male patients.

Ethical approval was obtained from the University of Otago Ethics Committee (H15/005), all methods were performed in accordance with the relevant guidelines and regulations, and all participants gave signed informed consent. Participants in the first two samples were reimbursed at a rate of NZ$15 per hour (above the minimum wage at the time). All patient participants received petrol vouchers (NZ$30 worth) in compensation for their time and travel costs including diagnostic examination as well as testing.

### Apparatus/materials

Participants were seated on an office chair, with task stimuli and questionnaires presented on an eye-level PC computer monitor (screen: 360 mm × 375 mm). Half the questionnaires were delivered before and half after the EEG task. The first half contained the trait items from the Spielberger State Trait Anxiety Inventory Y-form (STAI^44^; the Extraversion and Neuroticism items from the Eysenck Personality Questionnaire-Revised EPQ-R^49^; and the BIS items from the Behavioural Activation System/Behavioural Inhibition System scale BIS/BAS^50^. The second half contained the Anhedonia, Anxiousness, Depressivity, Emotional Lability, Intimacy Avoidance, Perseveration, Risk Taking, Restricted Affectivity, Separation Insecurity and Withdrawal scale items from the Personality Inventory of the DSM-5 PID5^40^. Questions about sleep and history of depression were also included. Participants also provided demographic information, including age, gender, and ethnicity. Of these measures, only the PID-5 Depressivity, PID-5 Anxiousness and STAI-T scales were analysed in this paper. STAI-T is a well-established measure derived from clinical severity and is thought to encompass elements of both depressivity and anxiousness^51–54^. We thought it important to compare obtained relationships between the newer depressivity, anxiousness and the older STAI-T scales with relevant electrophysiological factors. EEG was recorded (256 Hz sampling rate, 1–36 Hz bandpass) via a 32-channel Waveguard EEG cap (ANTneuro, Netherlands) from 18 channels of the 10–20 system: Fp1, Fpz, Fp2, F7, F3, Fz, F4, F8, T7, C3, Cz, C4, T8, P7, P3, Pz, P4, and P8 re-referenced to the average of A1 and A2 (mastoids). Resting EEG data were recorded in 1 minute blocks of eyes open (EO) or eyes closed (EC) for 8 minutes (in the order: EO, EC, EO, EC, EC, EO, EC, EO) or 10 minutes (alternating EO and EC). During this time, “OPEN” or “CLOSED” was displayed on the computer screen with the experimenter providing a verbal prompt when he word “OPEN” appeared. Prior to the start of testing the participants were informed that during the OPEN condition they could look anywhere they wished, including at the screen, but that they should try not to move their heads or bodies.

### Procedure

Participants provided signed informed consent then completed the first set of questionnaires (10–15 minutes). An appropriate sized EEG cap was fitted and electrode impedances reduced to <20 KΩ. Two different procedures were used: (1) Resting EEG recording followed by an SST (data not reported; 66 healthy participants, 48 anxiety patients) and (2) SST followed by resting EEG recording (33 healthy participants). The EEG cap was then disconnected and removed and the second set of questionnaires completed.

### Data processing and analysis

#### EEG pre-processing

EEG data were exported to EEGLAB^55^ and split into 1-second, non-overlapping epochs for data rejection. Epochs (256 samples) were visually inspected and deleted if they contained muscle or movement artefacts. Independent component analysis with ADJUST 1.1^56^ removed eye blink and eye movement artefact components to leave ‘clean’ EEG. The data were re-epoched into Hanning windowed, 50% overlapping, 2-second epochs. Two additional channels were then synthesised using raw EEG scores:

$${\rm{AF}}3=({\rm{F}}3+{\rm{Fp}}1)/2;{\rm{AF}}4=({\rm{F}}4+{\rm{Fp}}2)/2.$$

#### Alpha asymmetry

For fixed band alpha asymmetry we applied a Fourier transform, converted it to the power spectrum, and took an initial bandwidth of 6–12 Hz based on the ‘transition frequency’^57^ and analysed it both as a total (6–12 Hz) and split it into 3 sub-bands, low 2 (6–8 Hz), low 1 (8–10 Hz), high (10–12 Hz). The AA score was calculated for each homologous electrode pair (frontal: Fp2-Fp1, AF4-AF3, F4-F3, F8-F7; posterior: P4-P3, P8-P7) for each sub-band as:

$$AA=(ln\,(Right\,power)-ln\,(Left\,power))$$

This generated 18 separate AA measures in all.

#### Fractal dimension

Fractal dimension (FD), when applied to EEG signals, returns a fractional value between 1 and 2. The integers represent the topological dimensions of the EEG time series (a line, potentially 1-dimensional) relative to the space it is plotted in (a plane, 2-dimensional). The FD tells us how much of the 2-dimensional space our 1-dimensional line is taking up. A simple straight line will have a fractal dimension of 1 (taking up minimum space), while the more complex an EEG signal is, the more it will fill space and the more it will have a fractal dimension closer to 2 than 1.

There are a range of methods developed to calculate the FD of a signal. In this paper we used Higuchi’s Fractal Dimension (HFD) due to its level of accuracy^38^. HFD assesses multiple time series repeatedly subsampled from the original signal, recreating the original at a range of scales. The length of the curve for each subseries is calculated, and then averaged across sets. This process is repeated for different scales and plotted on a double logarithmic graph. HFD is the slope of the line formed by this graph. See Supplementary Materials for a more technical explanation of the method.

#### Statistical analysis

All statistical analysis used the IBM SPSS Statistics package (version 24). Comparison of the correlations of the independent healthy samples revealed no significant differences between them, and so they were pooled for analysis. Anxiety patient data are reported separately and combined with the healthy participants. Given the large number of possible ways of estimating AA and HFD we first carried out a set of simplifying analyses (Supplementary Material). With significant differences between estimation methods we chose the best method for each measure, otherwise we chose the condition most commonly used in the literature.

After these simplifying choices, the remaining variables were subjected to stepwise regressions predicting depressivity and anxiousness. AA or HFD predictors that survived the stepwise were regressed with each other to determine whether variance accounted for was shared or unique to the variable. PID-5 depressivity, PID-5 anxiousness and STAI-T were then used as predictors for each optimised EEG measure separately to see whether the variance accounted for was shared between the scales or was unique.

## Supplementary information

Supplementary Information

## Data Availability

The datasets analysed during the current study are available from the corresponding author on reasonable request.
