# Supplementary material for: Higuchi’s fractal dimension, but not frontal or posterior alpha asymmetry, predicts PID-5 anxiousness more than depressivity
Source: Sci Rep. 2019 Dec 23;9:19666. doi: 10.1038/s41598-019-56229-w (PMC6928148; doi:10.1038/s41598-019-56229-w)
Supplement: Supplementary file 1 — Supplementary Information [file 41598_2019_56229_MOESM1_ESM.pdf]

Higuchi's fractal dimension, but not frontal or posterior alpha asymmetry, predicts PID-5 anxiousness more than depressivity

Tame N. J. Kawe, Shabah M. Shadli, and Neil McNaughton*

Dept. of Psychology, University of Otago, Dunedin, New Zealand

Supplementary material

Higuchi's Fractal Dimension – method details

Higuchi's Fractal Dimension (Higuchi, 1988) is a method of calculating the Fractal Dimension (FD) of a time series. For EEG signals the HFD will return a value between 1 and 2 with a simple straight line delivering a value of 1 and higher values indicating increased complexity within the signal. A fractal is self-similar at all scales, large or small.

The HFD creates multiple time series by subsampling the signal repeatedly. This creates the original signal at a variety of different scales. The length of the curve for each new time series is then calculated and averaged across the sets. This is repeated for the different scales and plotted on a double logarithmic graph. The fractal dimension is the slope of this graph.

The HFD first generates new time series described as:

$$X_k^m;\ X(m),\ X(m+k),\ X(m\ +2k), \dots X\left(m\ +\ \left[\frac{N-m}{k}\right]\ \times k\right)\ \ (m=1,2,3,\dots,k), \quad (1)$$

Where X is the original time series, *m* indicates the initial time and *k* is the interval time. For each of the new time series X_k^m the length of the curve is calculated by:

$$L_m(k) = \left\{\left(\sum_{i=1}^{\left[\frac{N-m}{k}\right]} |X(m+ik) - X(m+(i-1)\cdot k|\right)\frac{N-1}{\left[\frac{N-m}{k}\right]\cdot k}\right\}/k \quad (2)$$

While the length of the curve for $k, L(k)$ is the average value over k sets of $L_m(k)$. If $L(k)$ is proportional to k^{-D} then the curve has the fractal dimension D. D is the slope of a least mean

squares straight line applied to $L(k)$ and k plotted on a double logarithmic graph for k ranging from 1 to $k_{\max}$.

$$\log(L(k)) \sim \log(\tfrac{1}{k}) \quad (3)$$

In the current study we used a $k_{\max}$ value of 30, this was determined by plotting HFD over a range of $k_{\max}$ and selecting the value at which the slope plateauxed.

Condition analysis to reduce the number of measures analysed

Experiments with resting EEG in the previous literature have tested people with their eyes open, eyes closed, or alternating between the two states. Eyes open would be expected to maintain a higher level of vigilance than eyes closed; and switching regularly between the two conditions would be expected to maintain a more constant average level of relaxation and also to prevent participants going to sleep. Preventing sleep is important as this would generate quite distinct EEG from the normal relaxed state. The effect of eye conditions was analysed as described below.

One-way, repeated measures ANOVAs were used to separately test the effects on average depressivity variance accounted for by each of AA eye condition, HFD eye condition and frequency band (after choice of eye condition, see below). Correlations with depressivity were converted to signed percent of variance (r^2 * 100, with the sign of r retained), to avoid the skew of the raw correlation scores while keeping the results easy to interpret. The different calculation methods (varying in choice of electrode pair used) for AA provided the source of error variance for the ANOVA.

RM Eye Condition – Alpha Asymmetry

Signed percent variance accounted for by AA and HFD were submitted to a one-way, repeated measures ANOVA separately for depressivity and anxiousness to assess gender (male, female) and eye condition (close, combined, open) effects. Mean values are presented in Table S1 and plotted in Figure S1, while within-subjects contrasts are in Table S2.

Table S1. Mean signed percent anxiousness and depressivity variance accounted for (averaged over bandwidths and/or electrode pairs with the signs of the individual correlations retained) for different AA and HFD eye conditions.

Gender	Eye Condition	AA		HFD	
		A	D	A	D
Female	Closed	1.3	1.3	6.4	3.9
	Combined	1.5	1.1	5.0	2.4
	Open	2.0	1.3	3.2	1.4
Male	Closed	1.1	2.2	8.3	3.6
	Combined	1.3	1.9	9.6	4.5
	Open	1.4	1.4	9.7	4.9
Whole Sample	Closed	1.2	1.8	7.4	3.7
	Combined	1.4	1.5	7.3	3.5
	Open	1.7	1.3	6.5	3.2

The highest percent variance values for AA appear in the open eye condition for anxiousness and the closed eye condition for depressivity; however these values are small and did not reach significance. There was also no significant difference in average percent variance between genders. For anxiousness, females may have had a higher scores than males in the open condition only (Eye[[quad] x gender[dev], $F(1,14) = 4.567$, $p = 0.051$). For depressivity in males, there was a steady decline from closed to open eye conditions while females showed some decline between the closed and combined conditions but then an increase between combined and open. As a result, the difference between the genders is non-linear, with no difference between them in the open condition (Eye[quad] x Gender[dev], $F(1,14) = 6.659$, $p = 0.022$). The highest values for both genders were obtained in the closed eye condition. In the absence of strong differences between the eye conditions (Eye[lin], $F(1,14) = 0.624$, $p = 0.443$) , the closed eye condition was selected for further analysis as it is the most commonly used in the previous literature.

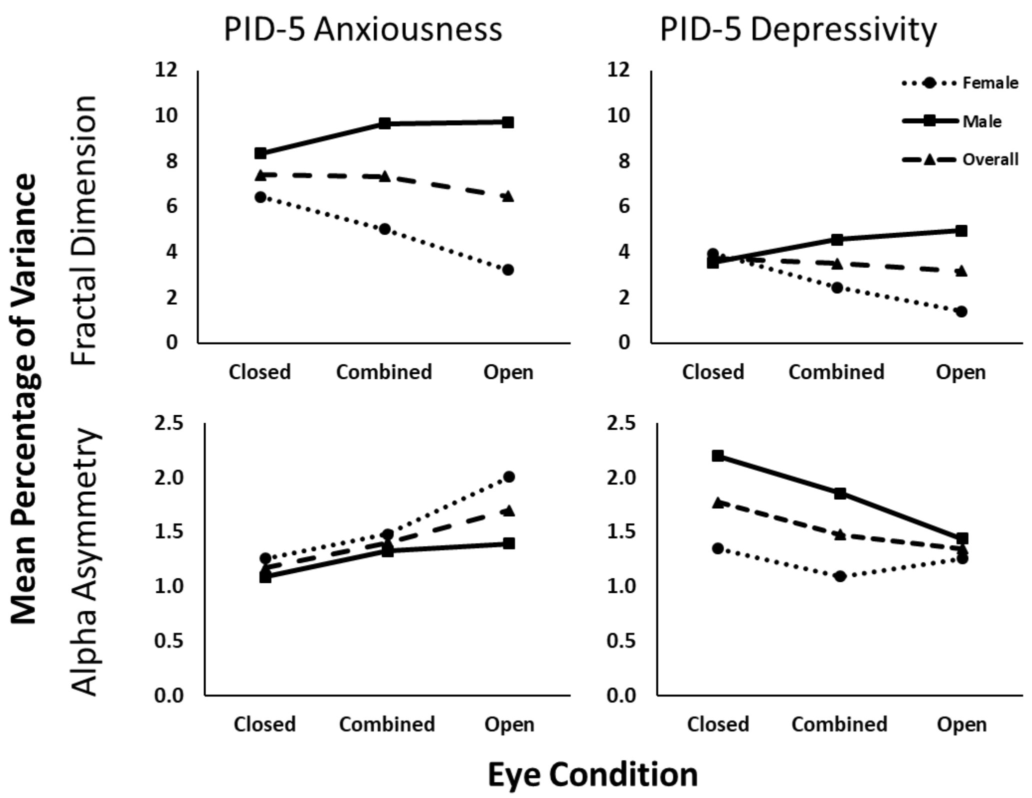

Figure S1. Mean signed percent variance accounted for by the correlations of fractal dimension or alpha asymmetry with anxiousness or depressivity vary with eye condition, and gender. The strongest relationships are for fractal dimension. Gender differences are generally greatest in the eyes open condition, except for the relation of alpha asymmetry to depressivity.

RM Eye Condition – HFD

The highest overall HFD percent variances were obtained in the closed eye condition. However, this varies by gender. In depressivity, male participants showed variance that increased from closed to open eye conditions, while female participants showed the opposite pattern (Eye[lin] x Gender[dev], $F(1,17) = 40.534$, $p < .001$). For anxiousness, a similar pattern can be seen. Male participants display an increase in variance from closed to open eye conditions while female participants display a decline (Eye[lin] x Gender[dev], $F(1,17) = 15.324$, $p = .001$). These differences average out when gender is not taken into account, leaving the closed eye condition with the

highest variances. Due to the higher proportion of female participants and the nominally higher values across the overall sample the closed eye condition was selected for further analysis.

Table S2. F statistics for contrasts extracted from repeated measures ANOVA for eye condition and gender.

			AA		HFD	
Measure	Condition	Polynomial	F	p	F	p
Anxiousness	Gender	Linear	.441	.517	12.858	.002
	Eye Condition	Linear	2.391	.144	3.857	.066
		Quadratic	.254	.622	42.47	<.001
	Eye x Gender	Linear	.447	.515	15.342	.001
		Quadratic	4.567	.051	11.928	.003
Depressivity	Gender	Linear	1.491	.242	7.255	.015
	Eye Condition	Linear	.624	.443	3.146	.094
		Quadratic	2.318	.150	.0548	.469
	Eye x Gender	Linear	.775	.393	40.534	<.001
		Quadratic	6.659	.022	23.487	<.001

RM Bandwidth

Table S3. Mean PID-5 depressivity and PID-5 anxiousness signed percent variance accounted for by alpha asymmetry in specified bands

Gender	Band	Measure	
		A	D
Whole sample	Low 2	1.5	1.2
	Low 1	1.3	2.4
	High	2.3	1.7
Female	Low 2	1.8	1.8
	Low 1	1.8	1.9
	High	2.4	0.3
Male	Low 2	1.1	0.5
	Low 1	0.7	3.0
	High	2.3	3.2

A repeated measures ANOVA comparing AA bands in the closed eye condition determined if retaining separate bands were necessary. Means can be seen in Table 3. A Band x Gender effect ($F(1,4) = 11.619$, $p = .027$) was seen for depressivity with male participants showing higher values in the High and Low 1 bands while female participants had low values in the high band. For anxiousness the highest values were seen in the high band, but differences between bands were not significant. Due to the Band x Gender effect seen for depressivity, bands were kept separate for further analysis.

HFD Factor Analysis

There was a high level of intercorrelation of HFD between channels. A principle components analysis initially identified 2 components with eigenvalues exceeding 1, a 3rd component was included to account for residual noise. The three components (see Fig. 2) appear to represent frontal, lateral and posterior aspects of HFD. The components appear functionally identical across gender although they differed in weighting. Female participants loaded most strongly onto the posterior, then the frontal, and finally the lateral component (eigenvalues: 13.663, 1.297, .797 respectively) Male participants displayed the opposite pattern: frontal, then posterior, and again finally lateral (eigenvalues: 14.667, .943, .762 respectively).

HFD scores were then combined into two measures reflecting the main components. Frontal (FP2, FPZ, FP1, F8, F4, FZ, F3, F7) and posterior channels (P8, P4, PZ, P3, P7) were each averaged to create a combined frontal and posterior HFD measure. The lateral component was excluded as it was of negligible value in both genders. As the topography of the components varied little by gender further analysis did not split results by gender.

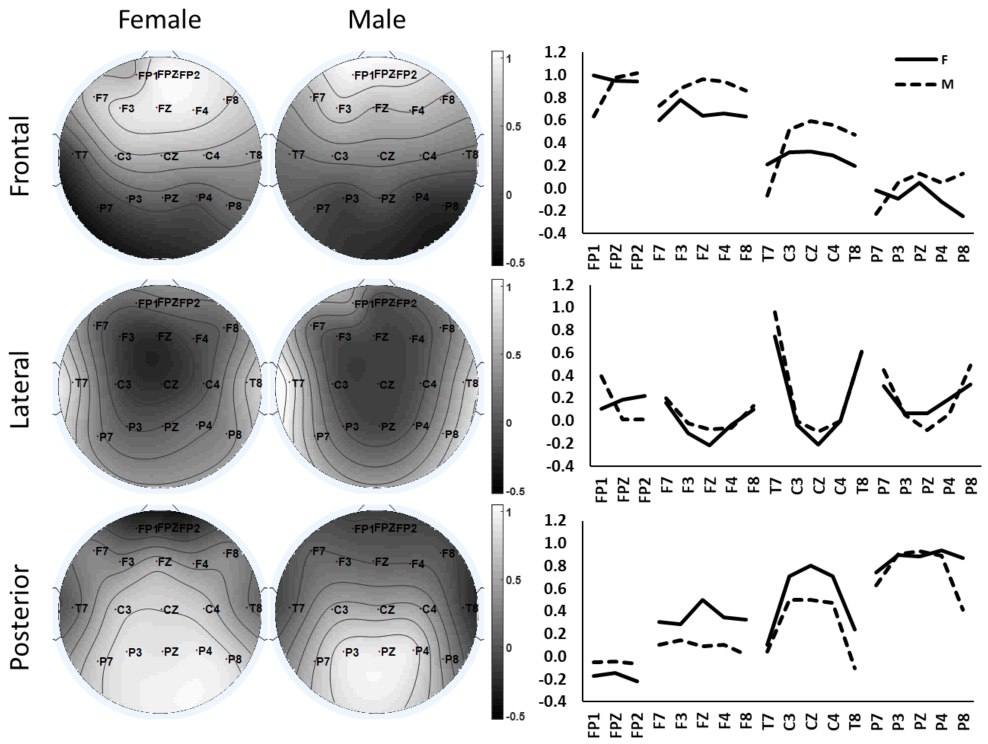

Figure S2. Heatmaps and associated line graphs displaying pattern matrices for selected principle components. The pattern matrices are labelled frontal, lateral and posterior given their distributions. Males loaded more on the frontal and females on the posterior components. The lateral component had the smallest eigenvalue (<1) for both genders and so was ignored in further analysis.
